# Harnessing cellulose-binding protein domains for the development of functionalized cellulose materials

**DOI:** 10.1186/s40643-024-00790-4

**Published:** 2024-07-25

**Authors:** Shaowei Li, Guodong Liu

**Affiliations:** 1 Taishan College, School of Life sciences, Shandong University, 72 Binhai Road, Qingdao, Shandong 266237 China; 2 State Key Laboratory of Microbial Technology, Shandong University, 72 Binhai Road, Qingdao, Shandong 266237 China

**Keywords:** Cellulosic materials, Cellulose-binding protein, Carbohydrate binding module, Material functionalization

## Abstract

Cellulosic materials are attracting increasing research interest because of their abundance, biocompatibility, and biodegradability, making them suitable in multiple industrial and medical applications. Functionalization of cellulose is usually required to improve or expand its properties to meet the requirements of different applications. Cellulose-binding domains (CBDs) found in various proteins have been shown to be powerful tools in the functionalization of cellulose materials. In this review, we firstly introduce the structural characteristics of commonly used CBDs belonging to carbohydrate-binding module families 1, 2 and 3. Then, we summarize four main kinds of methodologies for employing CBDs to modify cellulosic materials (i.e., CBD only, genetic fusion, non-covalent linkage and covalent linkage). Via different approaches, CBDs have been used to improve the material properties of cellulose, immobilize enzymes for biocatalysis, and design various detection tools. To achieve industrial applications, researches for lowering the production cost of CBDs, improving their performance (e.g., stability), and expanding their application scenarios are still in need.

**Graphical abstract:**

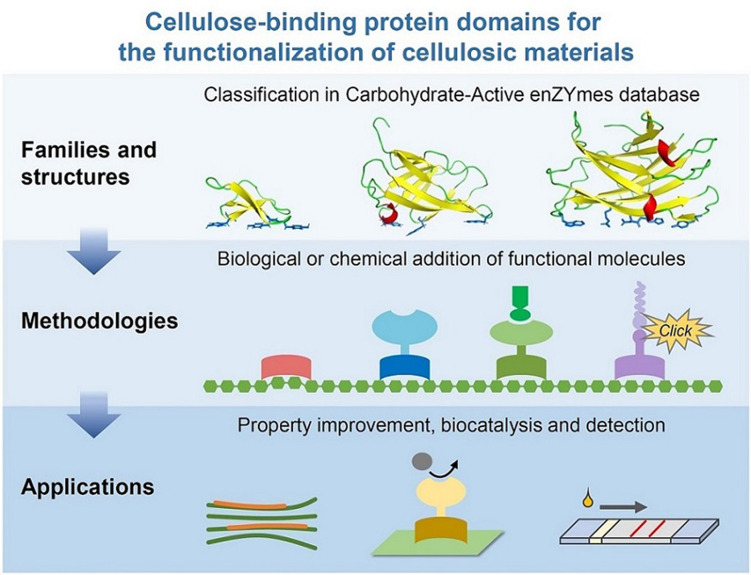

## Introduction

Cellulose is the most abundant biopolymer on Earth (Seddiqi et al. 2021). Owing to their stability, high-yield, low-cost, and renewable characteristics, cellulose materials are widely used in various industries, including papermaking, textiles, packaging, and medicine (Chen et al. 2022; Li et al. 2024a, b). With increasing demand for renewable resources and environmentally friendly materials, cellulose has attracted increasing attention as a biocompatible, biodegradable and widely sourced biomass material.

Cellulose is composed of β-1,4-linked d-glucose units (Heinze 2016), and the degree of polymerization varies among different cellulose materials (Hallac and Ragauskas 2011). The hydroxyl groups on the cellulose chain can form numerous intra- and intermolecular hydrogen bonds, resulting in a tightly ordered and insoluble crystalline structure. To enhance its solubility, thermoplasticity, hydrophobicity, and other desired properties, chemical modification of cellulose is often necessary. Each glucose monomer in the cellulose chain contains three hydroxyl groups that are susceptible to various chemical reactions, including oxidation, esterification, etherification, and graft copolymerization (Aziz et al. 2022; Heinze 2016). However, because cellulose easily decomposes under acidic or high-temperature conditions (Bartnik and Facey 2017), it is necessary to strictly control the pH and temperature of the chemical reactions to prevent hydrolysis and oxidation, thus obtaining more complete cellulose chains (Coseri 2017). In addition, some chemical reactions are characterized by low site selectivity, low reaction efficiency, and many side reactions, limiting their use in the improvement of cellulose materials (Wang et al. 2018).

Carbohydrate-binding modules (CBMs) are commonly found in carbohydrate-active enzymes and non-catalytic proteins. The CBMs can interact with carbohydrates through non-covalent bonds and bring linked catalytic protein domain(s) to the surface of the substrate to enhance enzyme activity (Sidar et al. 2020). Because of their high specificity, CBMs have been applied to develop technologies in diverse fields (Shoseyov et al. 2006). For example, recombinant CBMs can serve as tags for protein purification via affinity chromatography (Oliveira et al. 2015a, b). Cellulose-binding domains (CBDs) found in lignocellulose-degrading enzymes or related proteins (e.g., scaffolds of bacterial cellulosomes) were among the earliest studied CBMs (Linder and Teeri 1997). In terms of improving the performance of cellulose materials, CBDs have many advantages over other chemical methods, such as mild operating conditions, high specificity, reversibility, and diversity of functionalization forms. In this review we summarize the methods and applications of CBDs in the functionalization of cellulose materials. Future directions for the further development of highly efficient and low-cost materials based on CBDs are also discussed.

## Types and structures of CBDs

Based on structural similarities, CBMs are classified into 101 families in the Carbohydrate-Active enZymes (CAZy) database (as of April 2, 2024) (Drula et al. 2022). The modules in families 1, 2, 3, 4, 6, 8, 9, 10, 11, 16, 17, 28, 30, 37, 44, 46, 49, 59, 64, 65, 72, 76, 78, 79, 81, and 85 are reported to bind cellulose. Notably, the type of bound cellulose can differ among different CBM families; for example, the members of CBM1, CBM3, and CBM5 bind to crystalline cellulose, whereas those in CBM4 bind to amorphous cellulose. In addition, there are significant differences in the knowledge base of different CBM families. For example, 24 experimentally determined structures of CBM1 have been deposited in the RCSB PDB database (https://www.rcsb.org/). In contrast, only one structure each has been reported in the CBM9 and CBM10 families, and the cellulose-binding ability of the CBM72 family has been reported in only one case (Duan et al. 2016).

**Fig. 1:**
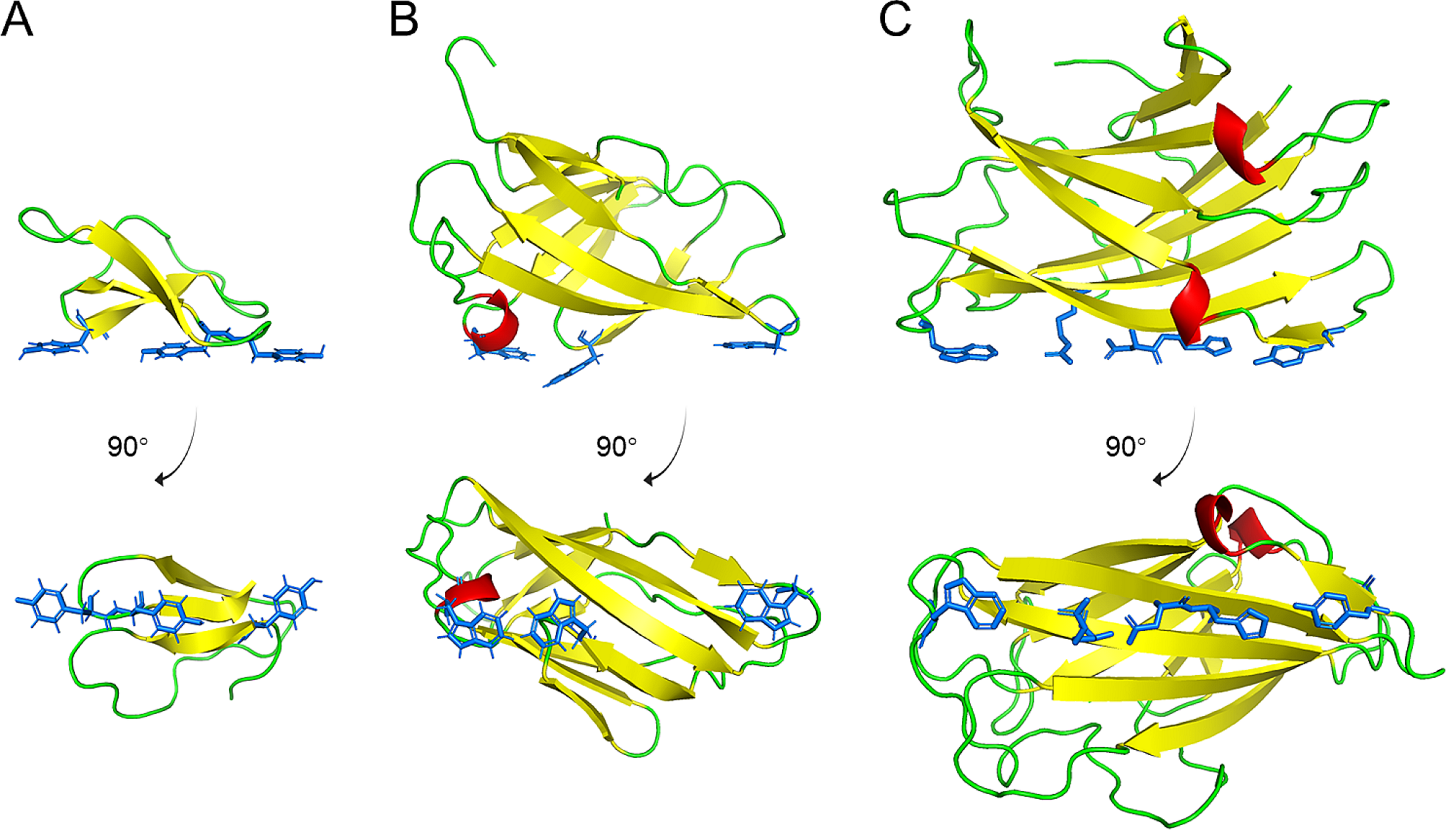
Structures of commonly used cellulose-binding domains (CBDs) in cellulose functionalization. Amino acid residues believed to interact with cellulose are shown in cyan color. (**A**) CBD in *T. reesei* cellobiohydrolase I belonging to CBM1 (PDB ID: 1CBH); (**B**) CBD in *C. fimi* exo-β-1,4-glucanase Cex belonging to CBM2 (PDB ID: 1EXG); (**C**) CBD in *C. thermocellum* (*Acetivibrio thermocellus*) cellulosomal-scaffolding protein belonging to CBM3 (PDB ID: 1NBC)

CBDs in the CBM families 1, 2 and 3 are mostly used for the functionalization of cellulose materials. CBM1 is the earliest discovered CBM family (Van Tilbeurgh et al. 1986). The first structure determined in this family was the CBD of cellobiohydrolase I in *Trichoderma reesei*, which contains 36 amino acids (Kraulis et al. 1989) (Fig. 1A). This domain has a wedge-like shape, with one side mainly hydrophilic and the other hydrophobic (Kraulis et al. 1989). Tyr5, Tyr31, and Tyr32, located on the flat and hydrophilic face, are believed to be directly involved in binding to cellulose (Shiiba et al. 2013). Two CBM2 domains were first discovered in the endo-β-1,4-glucanase CenA and exo-β-1,4-glucanase Cex from *Cellulomonas fimi* (Gilkes et al. 1988). The 110-residue CBD in Cex is rich in β-sheets and has a β-barrel fold (Xu et al. 1995) (Fig. 1B). The surface residues Trp17, Trp54, and Trp72 are considered to be important for binding to cellulose (McLean et al. 2000). The CBM3 domain was first identified in a cellulosomal-scaffolding protein of *Clostridium thermocellum* (Poole et al. 1992). This domain comprises approximately 150 amino acids and has a β-jelly roll structure similar to that of CBM2 (Tormo et al. 1996) (Fig. 1C). The β-sheets form two flat surfaces, and Trp118, Arg112, Asp56, His57, and Tyr67 on one surface are assumed to directly contact with the cellulose chain (Tormo et al. 1996). CBDs in the three families seem to share a conserved mechanism of interacting with crystalline cellulose through linearly arranged aromatic and polar residues. Notably, CBM3 domains feature the most extensive planar surface area, effectively engaging with three cellulose chains simultaneously.

## Methodologies for the functionalization of cellulose materials using CBDs

**Fig. 2:**
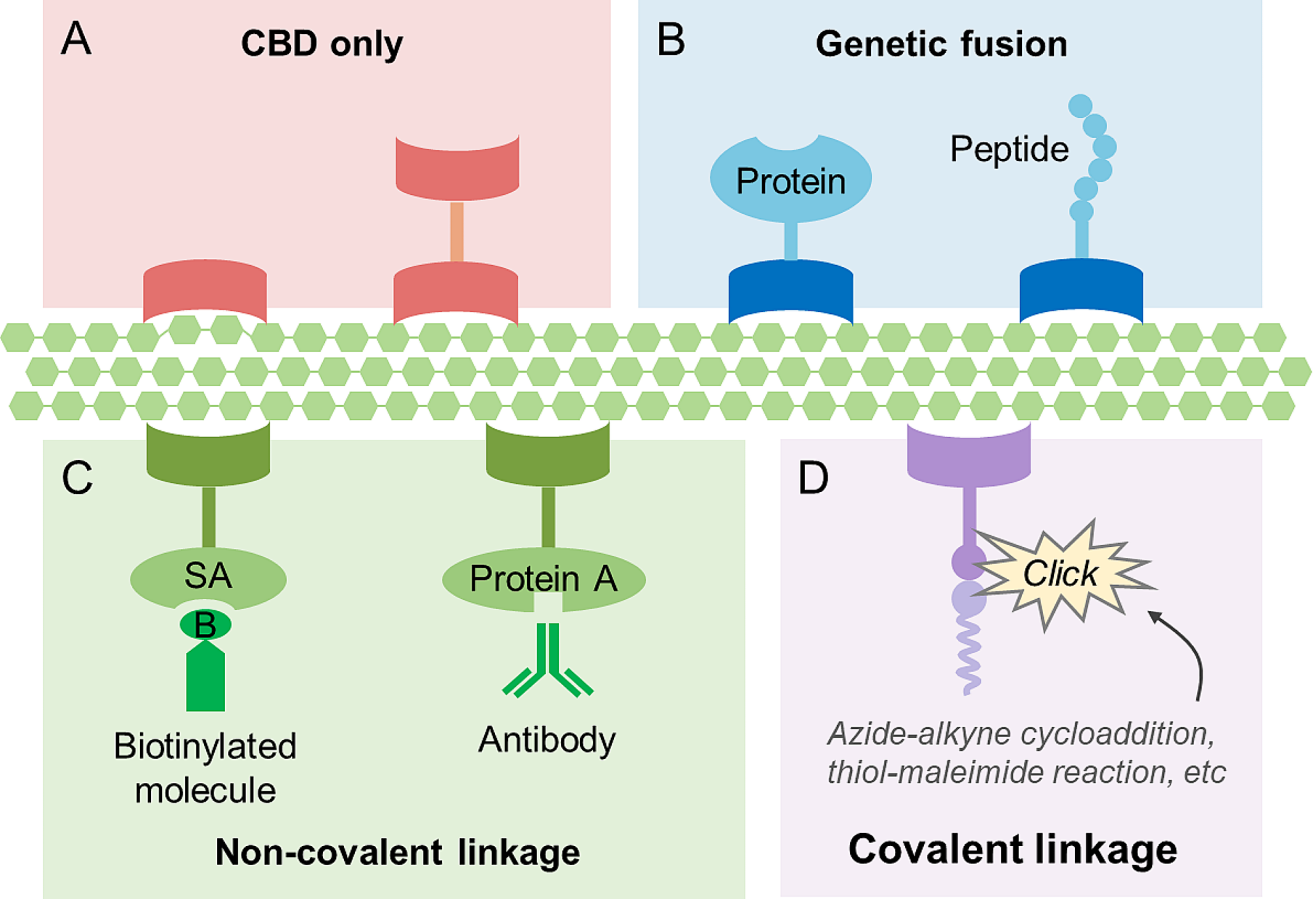
Different methods for constructing functionalized cellulose materials using CBDs. (**A**) Using single- or bi-modular CBDs only; (**B**) Fusion with proteins or peptides having inherent functions; (**C**) Non-covalent anchoring of molecules after fusion with peptides or proteins. SA, streptavidin; (**D**) Addition of molecules through covalent bonds

CBDs can be used directly to change the physical and chemical properties of cellulose (Fig. 2A; examples are given in the next section). However, in most cases, CBDs are used to modify cellulose by linking other molecules to its surface, thereby greatly extending its function (Barbosa et al. 2021; Yang et al. 2015). Molecules can be tethered to the cellulose surface via CBDs in different ways. First, functional proteins or peptides, such as enzymes and antimicrobial peptides, can be genetically fused to CBDs to improve the performance of materials or endow materials with new characteristics (Fig. 2B). Second, molecules can be indirectly linked to the CBDs, and consequently, to the cellulose surface through noncovalent interactions (Fig. 2C); for example, streptavidin can be fused with CBDs to provide a platform for binding of various biotinylated molecules (Pelus et al. 2021). Similarly, antibodies can be anchored to cellulose surface by using fusion constructs of CBD and staphylococcal protein A. Lastly, functional molecules can be anchored to CBDs by covalent bonds, usually through “click chemistry” strategies (Fig. 2D) (Geng et al. 2021). An azide group can be introduced into the CBD through the incorporation of a noncanonical amino acid, and functional molecules can then be added via azide-alkyne cycloaddition under physiological conditions (Agard et al. 2005). Alternatively, molecules can be conjugated to the CBD through cysteine-maleimide chemistry, which does not require the use of noncanonical amino acids (Barbosa et al. 2021; Pfaff et al. 2022). Additionally, primary amines on the surface of natural CBDs can be employed for the conjugation of molecules after the introduction of alkyne groups (Aïssa et al. 2019). The SpyCatcher-SpyTag system can also be used to construct CBD-containing complexes by forming covalent bonds (Griffo et al. 2019). Using the above strategies, various molecules (e.g., proteins, peptides, oligonucleotides, and small compounds) can be anchored to the surface of cellulose through the mediation of CBDs.

## Improving the properties of cellulose materials with CBDs

Some CBDs can disrupt the crystalline structure of cellulose even in the absence of a catalytic domain. Therefore, the use of CBDs alone could alter certain features of cellulose. As an example, CBM2 was mixed with cotton to enhance the affinity of cotton cellulose to dyes; however, the dyes were found to easily wash away under alkaline conditions (Cavaco-Paulo et al. 1999). In addition, CBDs have been used to improve the hydrophobicity, drainability, and strength of paper (Oliveira et al. 2015b; Pala et al. 2001). A recent study showed that incorporating CBM3 into bacterial cellulose can improve its strength and ductility under various conditions (Liu et al. 2023). Double CBDs connected by a linker peptide are believed to have a crosslinking effect on the fibers, which enhances the mechanical strength of the paper material (Levy et al. 2002). Nevertheless, CBDs belonging to different CBM families exhibit different improvements in the paper properties (Shi et al. 2014).

Fusing CBDs with other molecules greatly expands their potential to improve the properties of cellulose materials. These molecules include abiotic compounds, enzymes with surface modification activities, and proteins or peptides that can bind to other materials. One example is modification of the cellulose surface with polyethylene glycol-linked CBM2, which improves the redispersion of cellulose nanocrystals after drying and the stability of the suspension; this method can be used to prepare self-assembled nanoparticles from polysaccharides and proteins (Aïssa et al. 2019). By fusing a cutinase that hydrolyze acetyl groups to CBDs, the wettability and dyeability of cellulose acetate fibers are reportedly significantly improved after treatment, and the effect was greater than that obtained using cutinase only (Zhang et al. 2012). Silicatein, a silica-polymerizing enzyme, can form a silica layer on the cellulose surface when fused with CBM3. This fusion facilitates direct interaction between silicatein and cellulose, enabling the deposition of silica in proximity to cellulose fibers (Godigamuwa et al. 2020). In another study, CBM1 was fused to the class II hydrophobin HFBI, which was connected to graphene through hydrophobic interactions, achieving graphene fixation on nanofibrillated cellulose to enhance its mechanical properties (e.g., ultimate tensile strength) (Laaksonen et al. 2011). In the latter two studies, the authors revealed that CBDs can mediate the hybridization of organic and inorganic materials, providing new possibilities for improving material properties.

Combining CBM with other functional molecules can endow cellulose materials with new features. An antimicrobial hexapeptide was linked to CBM3 and fixed onto cellulose, which greatly improved the antibacterial activity of cellulose materials, thus offering the prospect of medical applications of cellulose (Barbosa et al. 2022). The fusion of lysozyme with CBM2 can also endow cellulosic materials with antibacterial activity (Abouhmad et al. 2016). The combination of CBM3 with the ZZ fragment derived from protein A (Nilsson et al. 1987), along with an anti-biotin antibody, was used to immobilize biotin-labeled gold nanoparticles (AuNPs) onto cellulose materials. With the aid of such CBM3-ZZ-antibody complexes, a uniform distribution of AuNPs on the cellulose surface was achieved. This approach holds promise for imparting novel optical, electronic, and chemical functionalities to cellulose-based substrates (Almeida et al. 2017). Moreover, by connecting CBDs with metallothionein or a hexa-histidine tag, cellulose has been adapted to remove toxic metal ions (Togo et al. 2020; Xiao et al. 2020). Similarly, macromolecules can be adsorbed and fixed onto cellulose materials. For example, mini-proteins targeting the receptor-binding domain of SARS-CoV-2 were fused with CBDs, which can be used to capture viral particles on masks, reducing the possibility of cell infection by 500 times (Navone et al. 2022).

## Immobilizing enzymes for biocatalysis with CBDs

The ability of CBDs to anchor fused enzymes to the cellulose surface makes cellulose a convenient support matrix for enzyme immobilization. Oriented adsorption of enzymes aided by CBDs has the advantages of lower activity loss and less protein aggregation. In addition, the procedures for the purification and immobilization of enzymes may be combined when CBD tags are used, and sometimes the tag can improve the soluble expression of the target enzymes (Liao et al. 2012). With immobilization on cellulose, enzymes can be easily recycled (Estevinho et al. 2018), and their stability and catalytic efficiency have been reported to be improved in many cases (Table 1). Notably, the effects of CBD fusion and cellulose immobilization on enzyme performance may vary depending on the type of CBDs. For example, different kinds of CBDs were genetically fused with *cis*-epoxysuccinic acid hydrolase and subsequently immobilized on cellulose to evaluate their catalytic efficiencies. By screening five CBDs from four CBM families, CBM30 from *C. thermocellum* was found to be the best “partner” of *cis*-epoxysuccinic acid hydrolase, with the immobilized fusion enzyme showing a 140% increase in catalytic efficiency compared with the free native enzyme (Wang et al. 2012).

**Table 1:** Examples of immobilization of enzymes onto cellulose by CBDs for biocatalysis

Enzyme	CBM Family	Cellulose type	Results	Reference
β-galactosidase	CBM2	Bacterial cellulose	Similar hydrolysis performance compared with free enzyme	Estevinho et al. 2018
Polyphosphate glucokinase	CBM3	Regenerated amorphous cellulose	Eightfold half-life time as compared with that without immobilization	Liao et al. 2012
*Cis*-epoxysuccinic acid hydrolase	CBM30	Microcrystalline cellulose Avicel PH-101	140% increase in *k*_cat_/*K*_m_; no activity loss after 20 times recycling	Wang et al. 2012
Phosphoglucose isomerase	CBM3	Regenerated amorphous cellulose	80-fold half-life time as compared with free enzyme	Myung et al. 2011
Carbonic anhydrase	CBM3	Microcrystalline cellulose	90% activity retained after 40 days continuous reaction	Razzak et al. 2020
β-galactosidase	CBM3	Alkaline-, acid-, or non-treated microcrystalline cellulose	Less inhibition by galactose; 53–64% hydrolysis ability retained after 40 reuse cycles	Gennari et al. 2022
β-galactosidase	CBM3	High crystallinity cellulose Sigmacell Type 50	Higher thermostability than free enzyme; over 30% activity retained after 9 reuse cycles	Wang et al. 2021

CBDs have also been used for the co-immobilization of enzymes on cellulose surfaces for multi-enzymatic cascade reactions. The reaction rate can be enhanced by the substrate channeling effect in such cascades (Wang et al. 2023). Inspired by the organization of bacterial cellulosomes, researchers have used CBM3, cohesins, and dockerins from this natural multi-enzyme machine to assemble and immobilize triosephosphate isomerase, aldolase, and fructose 1,6-biphasase onto cellulose. This method of enzyme complex construction has been found to not only reduce the workload of protein purification, but also increase the reaction rate by one order of magnitude compared to a mixture of free enzymes (You and Zhang 2013). Further development of cellulose-containing magnetic nanoparticles as a support material has allowed easier and more selective control of the reactions (Myung et al. 2013).

## Designing of detection tools with CBDs

**Fig. 3:**
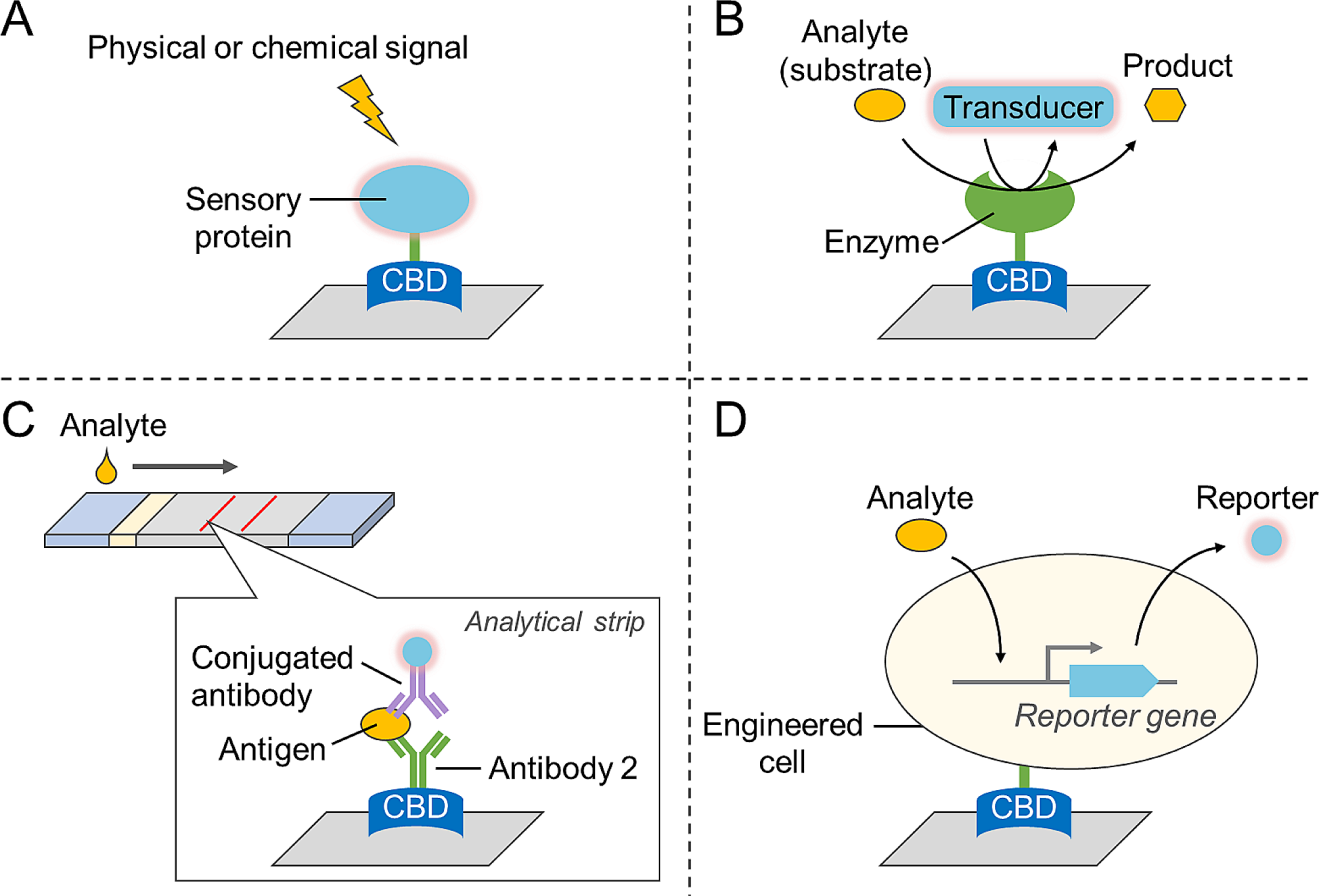
Applications of CBDs in cellulose-based detections. (**A**) Immobilization of sensory proteins (e.g., fluorescent proteins) on cellulosic materials; (**B**) Immobilization of enzymes; (**C**) immobilization of antibodies in lateral flow assay; (**D**) immobilization of whole-cell biosensors

When linked to sensory molecules or enzymes, CBDs can be used in the detection of substances or physicochemical signals (Fig. 3). For example, a pH-sensitive enhanced cyan fluorescence protein was fused to CBM2 to monitor the extracellular pH in live tissues growing on cellulose scaffolds using fluorescence lifetime imaging microscopy (O’Donnell et al. 2018).

To construct enzyme-based biosensor devices, enzymes are usually fixed on electrodes via random immobilization methods (e.g., glutaraldehyde crosslinking), which may lead to decreased enzyme activity and low sensitivity. The oriented immobilization of proteins on cellulose using CBDs represents a significant advancement in the development of high-performance biosensor devices. This method enables precise control over protein orientation and density on the cellulose surface, thereby enhancing the sensitivity, specificity, and stability of biosensors for various analytical applications. Researchers have immobilized glucose oxidase onto a cellulose-based electrode using CBM2, which enabled the development of a glucose biosensor that responded linearly to glucose concentration changes over a large range with high stability and repeatability (Gong et al. 2021). Similarly, an FAD-dependent glucose dehydrogenase was fixed on a nanocellulose modified electrode to detect glucose with high sensitivity and stability (Han et al. 2021).

After fusion with antibodies, CBDs can be used to develop devices for detecting corresponding antigens. The lateral flow assay (LFA) is an important method for point-of-care testing and is characterized by rapid detection, portability, and ease of operation. Nevertheless, the sensitivity and specificity of LFA are still lower than those of enzyme-linked immunosorbent assay (ELISA) and PCR methods (Liu et al. 2021). Nitrocellulose membranes for the physical adsorption of antibodies are commonly used as analytical strips in LFA, which have the drawback of lowering antibody activity due to random adsorption. The interaction between CBDs and cellulose has been employed to develop cellulose-based LFAs, in which antibodies are directly or indirectly immobilized on strips with the aid of CBDs (Table 2). CBDs can help control the orientation of antibodies on strips, thereby improving the sensitivity of detection. In addition, the replacement of nitrocellulose with cellulose is expected to reduce the manufacturing costs of LFAs.

**Table 2:** Applications of CBD-cellulose interactions in lateral flow assays

Target molecule	CBM Family	Fusion partner of CBD	Analytical strip	Reference
Prostate-specific antigen	CBM1, CBM3	Antibody-binding B and C domains of Protein A	Cellulose membrane	Yang et al. 2021
Human gonadotropin or SARS-CoV-2	CBM3	Full-length antibodies or single chain variable fragments	Lab-engineered cellulose-papers	Elter et al. 2021
Cystatin C	CBM3	ZZ domain	Nitrocellulose membrane coated with cellulose nanofibers	Natarajan et al. 2022
DNA	CBM3	ZZ domain	Chromatography paper	Rosa et al. 2014

This strategy of antibody immobilization on cellulose has also been used for DNA detection. In an earlier report, a CBM3-ZZ fragment fusion product was used to anchor an anti-biotin antibody to cellulose, which captured DNA hybrids formed by biotin-labeled targets and fluorescein-labeled probes. The detection results were reflected by fluorescence signals (Rosa et al. 2014). The system was characterized using fluorescence correlation spectroscopy to further validate its effectiveness (Rosa et al. 2017). Fluorescence correlation spectroscopy provides detailed insights into the dynamics and interactions of biomolecules immobilized on the cellulose surface, confirming the reliability and functionality of the detection system. When fluorescein in the above system was replaced with AuNPs, the detection results could be directly observed with the naked eye (Rosa et al. 2019).

When CBDs are connected or integrated with living organisms, such as bacteria, their detection range is further expanded. CBM2 was fused to the cell-surface protein OmpA and expressed in *Escherichia coli*, enabling it to bind to cellulose. Using this platform, a live bacterial sensor for L-arabinose based on fluorescence intensity was constructed (Long et al. 2021). In another study, *E. coli*-specific phage T7 was engineered to express chimeric proteins comprising CBM2 and reporter enzymes (luciferase or alkaline phosphatase). These phages were used to infect and lyse *E. coli* cells, releasing reporter enzymes onto the cellulose membrane or magnetic cellulose particles for enzymatic reactions and signal production. This method of bacterial detection has the advantages of being both rapid and user-friendly (Hinkley et al. 2018; Singh et al. 2019).

## Prospects

The specific affinity of CBDs for cellulose has great potential not only for cellulose modification, but also for immobilizing enzymes and other molecules on cellulosic materials. In the near future, CBDs are expected to be applied in medical diagnosis field. With the increasing demand for sustainable materials, CBDs are expected to have broader and more profound application prospects in multiple fields. In particular, the biocompatibility of cellulose materials and at least some CBDs is beneficial for in vivo biomedical applications (Żebrowska et al. 2024). Nevertheless, the applications of CBDs summarized in this review are still in the laboratory stage and there is still a lot of room for improvement in their performance and cost effectiveness. The main challenges and opportunities are described as follows.

First, CBD-based fusion proteins are mainly produced in *E. coli*; the relatively high costs of induced protein production, cell lysis, and protein purification may hinder their application in bulk materials. Secreted production in well-established industrial protein hosts (e.g., *Bacillus subtilis* and *Pichia pastoris* secrete tens of grams of protein per liter) and the selection of thermostable proteins with long half-lives are expected to lower the costs of industrial application of such fusion proteins.

Second, protein engineering strategies can be used to generate high-performance CBDs that satisfy the requirements of more application scenarios (e.g., higher affinity, higher stability, and controlled desorption). In terms of directed evolution, a mutant library of natural CBDs can be constructed and subjected to high-throughput screening. This strategy has been successfully used to develop plastic-binding peptides based on an *E. coli* cell surface display screening system (Apitius et al. 2019). With the rapid development of structural bioinformatics, rational design and machine learning techniques are expected to effectively accelerate the development of CBDs with new characteristics.

Third, in most studies on the functionalization of cellulosic materials, researchers have chosen to use CBDs belonging to CBM families 1, 2, and 3, particularly CBM3. CBDs from other CBM families as well as cellulose-binding peptides of other origins (Qi et al. 2008) are worth testing for their performance. In addition, CBDs of different structures may differ in terms of substrate specificity. For example, fluorescently-tagged CBM2 and CBM17 were used to detect the spatial distribution of crystalline and paracrystalline cellulose in cellulosic materials, respectively (Novy et al. 2020). By combining different types of CBDs and lithography technologies, patterning of cellulosic materials may be achieved to offer competitive advantages for applications (Wolfberger et al. 2015).

Lastly, some CBDs have been reported to bind to other polymer materials, such as chitin and synthetic plastics (Ekborg et al. 2007; Rennison et al. 2023). The degradation of chitin and polyethylene terephthalate was significantly enhanced by fusing CBDs with the corresponding hydrolytic enzymes (Limón et al. 2001; Ribitsch et al. 2013). With a deeper understanding and engineering of the interactions between CBDs and polymers, the application of CBDs in the functionalization of materials can be expanded.

## Data Availability

Data sharing not applicable to this article as no data sets were generated or analyzed during the current study.

## Abbreviations

AuNP: Gold nanoparticle
CBD: Cellulose-binding domain
CBM: Carbohydrate-binding module
ELISA: Enzyme-linked immunosorbent assay
LFA: Lateral flow assay

## References

[CR1] Abouhmad A, Mamo G, Dishisha T, Amin MA, Hatti-Kaul R (2016) T4 lysozyme fused with cellulose-binding module for antimicrobial cellulosic wound dressing materials. J Appl Microbiol 121(1):115–125 27028513 10.1111/jam.13146

[CR2] Agard NJ, Prescher JA, Bertozzi CR (2005) A strain-promoted [3 + 2] azide-alkyne cycloaddition for covalent modification of biomolecules in living systems. J Am Chem Soc 127(31):11196 10.1021/ja059912x 15547999

[CR3] Aïssa K, Karaaslan MA, Renneckar S, Saddler JN (2019) Functionalizing cellulose nanocrystals with click modifiable carbohydrate-binding modules. Biomacromolecules 20(8):3087–3093 31260278 10.1021/acs.biomac.9b00646

[CR4] Almeida A, Rosa AMM, Azevedo AM, Prazeres DMF (2017) A biomolecular recognition approach for the functionalization of cellulose with gold nanoparticles. J Mol Recognit 30(9):e2634 10.1002/jmr.2634 28417509

[CR5] Apitius L, Rübsam K, Jakesch C, Jakob F, Schwaneberg U (2019) Ultrahigh-throughput screening system for directed polymer binding peptide evolution. Biotechnol Bioeng 116(8):1856–1867 30982949 10.1002/bit.26990

[CR6] Aziz T, Farid A, Haq F, Kiran M, Ullah A, Zhang K, Li C, Ghazanfar S, Sun H, Ullah R, Ali A, Muzammal M, Shah M, Akhtar N, Selim S, Hagagy N, Samy M, Al Jaouni SK (2022) A review on the modification of cellulose and its applications. Polymers 14(15):3206 35956720 10.3390/polym14153206 PMC9371096

[CR8] Barbosa M, Simões H, Prazeres DMF (2021) Functionalization of cellulose-based hydrogels with bi-functional fusion proteins containing carbohydrate-binding modules. Materials 14(12):3175 34207652 10.3390/ma14123175 PMC8227779

[CR7] Barbosa M, Simões H, Pinto SN, Macedo AS, Fonte P, Prazeres DMF (2022) Fusions of a carbohydrate binding module with the small cationic hexapeptide RWRWRW confer antimicrobial properties to cellulose-based materials. Acta Biomater 143:216–232 35257951 10.1016/j.actbio.2022.02.042

[CR9] Bartnik M, Facey PC (2017) Chapter 8 - glycosides. In: Badal S, Delgoda R (eds) Pharmacognosy. Academic, Boston

[CR10] Cavaco-Paulo A, Morgado J, Andreaus J, Kilburn D (1999) Interactions of cotton with CBD peptides. Enzyme Microb Technol 25(8–9):639–643 10.1016/S0141-0229(99)00101-5

[CR11] Chen C, Xi Y, Weng Y (2022) Recent advances in cellulose-based hydrogels for tissue engineering applications. Polymers 14(16):3335 36015592 10.3390/polym14163335 PMC9415052

[CR12] Coseri S (2017) Cellulose: to depolymerize… or not to? Biotechnol Adv 35(2):251–266 28095321 10.1016/j.biotechadv.2017.01.002

[CR13] Drula E, Garron M-L, Dogan S, Lombard V, Henrissat B, Terrapon N (2022) The carbohydrate-active enzyme database: functions and literature. Nucleic Acids Res 50(D1):D571–D577 34850161 10.1093/nar/gkab1045 PMC8728194

[CR14] Duan C-J, Feng Y-L, Cao Q-L, Huang M-Y, Feng J-X (2016) Identification of a novel family of carbohydrate-binding modules with broad ligand specificity. Sci Rep 6(1):19392 26765840 10.1038/srep19392 PMC4725902

[CR15] Ekborg NA, Morrill W, Burgoyne AM, Li L, Distell DL (2007) CelAB, a multifunctional cellulase encoded by T7902, a culturable symbiont isolated from the wood-boring marine bivalve. Appl Environ Microb 73(23):7785–7788 10.1128/AEM.00876-07 PMC2168062 17933945

[CR16] Elter A, Bock T, Spiehl D, Russo G, Hinz SC, Bitsch S, Baum E, Langhans M, Meckel T, Dörsam E, Kolmar H, Schwall G (2021) Carbohydrate binding module-fused antibodies improve the performance of cellulose-based lateral flow immunoassays. Sci Rep 11(1):7880 33846482 10.1038/s41598-021-87072-7 PMC8042022

[CR17] Estevinho BN, Samaniego N, Talens-Perales D, Fabra MJ, López-Rubio A, Polaina J, Marín-Navarro J (2018) Development of enzymatically-active bacterial cellulose membranes through stable immobilization of an engineered β-galactosidase. Int J Biol Macromol 115:476–482 29678790 10.1016/j.ijbiomac.2018.04.081

[CR18] Geng Z, Shin JJ, Xi Y, Hawker CJ (2021) Click chemistry strategies for the accelerated synthesis of functional macromolecules. J Polym Sci 59(11):963–1042 10.1002/pol.20210126

[CR19] Gennari A, Simon R, Sperotto NDM, Bizarro CV, Basso LA, Machado P, Benvenutti EV, Renard G, Chies JM, Volpato G, de Volken CF (2022) Application of cellulosic materials as supports for single-step purification and immobilization of a recombinant β-galactosidase via cellulose-binding domain. Int J Biol Macromol 199:307–317 35007635 10.1016/j.ijbiomac.2022.01.006

[CR20] Gilkes NR, Warren RA, Miller RC, Kilburn DG (1988) Precise excision of the cellulose binding domains from two *Cellulomonas fimi* cellulases by a homologous protease and the effect on catalysis. J Biol Chem 263(21):10401–10407 3134347 10.1016/S0021-9258(19)81530-2

[CR21] Godigamuwa K, Nakashima K, Okamoto J, Kawasaki S (2020) Biological route to fabricate silica on cellulose using immobilized silicatein fused with a carbohydrate-binding module. Biomacromolecules 21(7):2922–2928 32543179 10.1021/acs.biomac.0c00730

[CR22] Gong W, Han Q, Chen Y, Wang B, Shi J, Wang L, Cai L, Meng Q, Zhang Z, Liu Q, Yang Y, Yang J, Zheng L, Li Y, Ma Y (2021) A glucose biosensor based on glucose oxidase fused to a carbohydrate binding module family 2 tag that specifically binds to the cellulose-modified electrode. Enzyme Microb Technol 150:109869 34489028 10.1016/j.enzmictec.2021.109869

[CR23] Griffo A, Rooijakkers BJM, Hähl H, Jacobs K, Linder MB, Laaksonen P (2019) Binding forces of cellulose binding modules on cellulosic nanomaterials. Biomacromolecules 20(2):769–777 30657665 10.1021/acs.biomac.8b01346 PMC6727214

[CR24] Hallac BB, Ragauskas AJ (2011) Analyzing cellulose degree of polymerization and its relevancy to cellulosic ethanol. Biofuel Bioprod Bior 5(2):215–225 10.1002/bbb.269

[CR25] Han Q, Gong W, Zhang Z, Wang L, Wang B, Cai L, Meng Q, Li Y, Liu Q, Yang Y, Zheng L, Ma Y (2021) Orientated immobilization of FAD-dependent glucose dehydrogenase on electrode by carbohydrate-binding module fusion for efficient glucose assay. Int J Mol Sci 22(11):5529 34073858 10.3390/ijms22115529 PMC8197230

[CR26] Heinze T (2016) Cellulose: structure and Properties. In: Rojas OJ (ed) Cellulose chemistry and properties: fibers, nanocelluloses and advanced materials. Springer International Publishing, Cham

[CR27] Hinkley TC, Singh S, Garing S, Le Ny A-LM, Nichols KP, Peters JE, Talbert JN, Nugen SR (2018) A phage-based assay for the rapid, quantitative, and single CFU visualization of *E. Coli* (ECOR #13) in drinking water. Sci Rep 8(1):14630 30279488 10.1038/s41598-018-33097-4 PMC6168599

[CR28] Kraulis PJ, Clore GM, Nilges M, Jones TA, Pettersson G, Knowles J, Gronenborn AM (1989) Determination of the three-dimensional solution structure of the C-terminal domain of cellobiohydrolase I from *Trichoderma reesei*. A study using nuclear magnetic resonance and hybrid distance geometry-dynamical simulated annealing. Biochemistry 28(18):7241–7257 2554967 10.1021/bi00444a016

[CR29] Laaksonen P, Walther A, Malho JM, Kainlauri M, Ikkala O, Linder MB (2011) Genetic engineering of biomimetic nanocomposites: diblock proteins, graphene, and nanofibrillated cellulose. Angew Chem Int Edit 50(37):8688–8691 10.1002/anie.201102973 21887760

[CR30] Levy I, Nussinovitch A, Shpigel E, Shoseyov O (2002) Recombinant cellulose crosslinking protein: a novel paper-modification biomaterial. Cellulose 9(1):91–98 10.1023/A:1015848701029

[CR31] Li X, Wan C, Tao T, Chai H, Huang Q, Chai Y, Wu Y (2024a) An overview of the development status and applications of cellulose-based functional materials. Cellulose 31(1):61–99 10.1007/s10570-023-05616-8

[CR32] Li Z, Waghmare PR, Dijkhuizen L, Meng X, Liu W (2024b) Research advances on the consolidated bioprocessing of lignocellulosic biomass. Eng Microbiol 4(2):100139 10.1016/j.engmic.2024.100139 PMC11611046 39629327

[CR33] Liao H, Myung S, Zhang YHP (2012) One-step purification and immobilization of thermophilic polyphosphate glucokinase from *Thermobifida fusca* YX: glucose-6-phosphate generation without ATP. Appl Microbiol Biotech 93(3):1109–1117 10.1007/s00253-011-3458-1 21766194

[CR34] Limón MC, Margolles-Clark E, Benítez T, Penttilä M (2001) Addition of substrate-binding domains increases substrate-binding capacity and specific activity of a chitinase from *Trichoderma Harzianum*. FEMS Microbiol Lett 198(1):57–63 11325554 10.1016/S0378-1097(01)00124-0

[CR35] Linder M, Teeri TT (1997) The roles and function of cellulose-binding domains. J Biotechnol 57(1):15–28 10.1016/S0168-1656(97)00087-4 9335165

[CR37] Liu Y, Zhan L, Qin Z, Sackrison J, Bischof JC (2021) Ultrasensitive and highly specific lateral flow assays for point-of-care diagnosis. ACS Nano 15(3):3593–3611 33607867 10.1021/acsnano.0c10035

[CR36] Liu Y, Ran Q, Guo J, Zhu W, Bushra R, Duan X, Huang Y, Jiang Z, Khan MR, Jin Y, Xiao H, Song J (2023) In-situ CBM3-modified bacterial cellulose film with improved mechanical properties. Int J Biol Macromol 243:125193 37285886 10.1016/j.ijbiomac.2023.125193

[CR38] Long L, Hu Y, Xie L, Sun F, Xu Z, Hu J (2021) Constructing a bacterial cellulose-based bacterial sensor platform by enhancing cell affinity via a surface-exposed carbohydrate binding module. Green Chem 23(23):9600–9609 10.1039/D1GC03097C

[CR39] McLean BW, Bray MR, Boraston AB, Gilkes NR, Haynes CA, Kilburn DG (2000) Analysis of binding of the family 2a carbohydrate-binding module from *Cellulomonas fimi* xylanase 10A to cellulose: specificity and identification of functionally important amino acid residues. Protein Eng Des Sel 13(11):801–809 10.1093/protein/13.11.801 11161112

[CR41] Myung S, Zhang X-Z, Zhang Y-HP (2011) Ultra-stable phosphoglucose isomerase through immobilization of cellulose-binding module-tagged thermophilic enzyme on low-cost high-capacity cellulosic adsorbent. Biotechnol Progr 27:969–975 10.1002/btpr.606 21630486

[CR40] Myung S, You C, Zhang Y-HP (2013) Recyclable cellulose-containing magnetic nanoparticles: immobilization of cellulose-binding module-tagged proteins and a synthetic metabolon featuring substrate channeling. J Mater Chem B 1(35):4419–4427 32261114 10.1039/C3TB20482K

[CR42] Natarajan S, Joseph J, França Prazeres DM (2022) Exploring carbohydrate binding module fusions and fab fragments in a cellulose-based lateral flow immunoassay for detection of cystatin C. Sci Rep 12(1):5478 35361862 10.1038/s41598-022-09454-9 PMC8970072

[CR43] Navone L, Moffitt K, Johnston WA, Mercer T, Cooper C, Spann K, Speight RE (2022) Bioengineered textiles with peptide binders that capture SARS-CoV-2 viral particles. Commun Mater 3(1):54 35991518 10.1038/s43246-022-00278-8 PMC9376897

[CR44] Nilsson B, Moks T, Jansson B, Abrahmsén L, Elmblad A, Holmgren E, Henrichson C, Jones TA, Uhlén M (1987) A synthetic IgG-binding domain based on staphylococcal protein A. Protein Eng Des Sel 1(2):107–113 10.1093/protein/1.2.107 3507693

[CR45] Novy V, Nielsen F, Olsson J, Aïssa K, Saddler JN, Wallberg O, Galbe M (2020) Elucidation of changes in cellulose ultrastructure and accessibility in hardwood fractionation processes with carbohydrate binding modules. ACS Sustain Chem Eng 8(17):6767–6776 32391215 10.1021/acssuschemeng.9b07589 PMC7202243

[CR46] O’Donnell N, Okkelman IA, Timashev P, Gromovykh TI, Papkovsky DB, Dmitriev RI (2018) Cellulose-based scaffolds for fluorescence lifetime imaging-assisted tissue engineering. Acta Biomater 80:85–96 30261339 10.1016/j.actbio.2018.09.034

[CR47] Oliveira C, Carvalho V, Domingues L, Gama FM (2015a) Recombinant CBM-fusion technology — applications overview. Biotechnol Adv 33(3):358–369 25689072 10.1016/j.biotechadv.2015.02.006

[CR48] Oliveira C, Sepúlveda G, Aguiar TQ, Gama FM, Domingues L (2015b) Modification of paper properties using carbohydrate-binding module 3 from the *Clostridium thermocellum* CipA scaffolding protein produced in *Pichia pastoris*: elucidation of the glycosylation effect. Cellulose 22(4):2755–2765 10.1007/s10570-015-0655-6

[CR49] Pala H, Lemos MA, Mota M, Gama FM (2001) Enzymatic upgrade of old paperboard containers. Enzyme Microb Technol 29(4):274–279 10.1016/S0141-0229(01)00380-5

[CR50] Pelus A, Bordes G, Barbe S, Bouchiba Y, Burnard C, Cortés J, Enjalbert B, Esque J, Estaña A, Fauré R, Henras AK, Heux S, Le Men C, Millard P, Nouaille S, Pérochon J, Toanen M, Truan G, Verdier A, Wagner C, Romeo Y, Montanier CY (2021) A tripartite carbohydrate-binding module to functionalize cellulose nanocrystals. Biomater Sci 9(22):7444–7455 34647546 10.1039/D1BM01156A

[CR51] Pfaff SA, Wang X, Wagner ER, Wilson LA, Kiemle SN, Cosgrove DJ (2022) Detecting the orientation of newly-deposited crystalline cellulose with fluorescent CBM3. Cell Surf 8:100089 36426175 10.1016/j.tcsw.2022.100089 PMC9678952

[CR52] Poole DM, Morag E, Lamed R, Bayer EA, Hazlewood GP, Gilbert HJ (1992) Identification of the cellulose-binding domain of the cellulosome subunit S1 from *Clostridium thermocellum* YS. FEMS Microbiol Lett 99(2–3):181–186 10.1111/j.1574-6968.1992.tb05563.x 1490597

[CR53] Qi M, O’Brien JP, Yang JJ (2008) A recombinant triblock protein polymer with dispersant and binding properties for digital printing. Biopolymers 90(1):28–36 17972282 10.1002/bip.20878

[CR54] Razzak MA, Lee DW, Lee J, Hwang I (2020) Overexpression and purification of gracilariopsis chorda carbonic anhydrase (GcCAα3) in *Nicotiana Benthamiana*, and its immobilization and use in CO_2_ hydration reactions. Front Plant Sci 11:563721 33329625 10.3389/fpls.2020.563721 PMC7717956

[CR55] Rennison AP, Westh P, Møller MS (2023) Protein-plastic interactions: the driving forces behind the high affinity of a carbohydrate-binding module for polyethylene terephthalate. Sci Total Environ 870:161948 36739021 10.1016/j.scitotenv.2023.161948

[CR56] Ribitsch D, Yebra AO, Zitzenbacher S, Wu J, Nowitsch S, Steinkellner G, Greimel K, Doliska A, Oberdorfer G, Gruber CC, Gruber K, Schwab H, Stana-Kleinschek K, Acero EH, Guebitz GM (2013) Fusion of binding domains to *Thermobifida cellulosilytica* cutinase to tune sorption characteristics and enhancing PET hydrolysis. Biomacromolecules 14(6):1769–1776 23718548 10.1021/bm400140u

[CR57] Rosa AMM, Louro AF, Martins SAM, Inácio J, Azevedo AM, Prazeres DMF (2014) Capture and detection of DNA hybrids on paper via the anchoring of antibodies with fusions of carbohydrate binding modules and ZZ-domains. Anal Chem 86(9):4340–4347 24716740 10.1021/ac5001288

[CR59] Rosa AMM, Prazeres DMF, Paulo PMR (2017) Fluorescence correlation spectroscopy study of the complexation of DNA hybrids, IgG antibody, and a chimeric protein of IgG-binding ZZ domains fused with a carbohydrate binding module. Phys Chem Chem Phys 19(25):16606–16614 28616941 10.1039/C7CP00662D

[CR58] Rosa AMM, Nazar MR, Prazeres DMF (2019) Colorimetric detection of DNA strands on cellulose microparticles using ZZ-CBM fusions and gold nanoparticles. Biotechnol J 14(8):e1800590 31144775 10.1002/biot.201800590

[CR60] Seddiqi H, Oliaei E, Honarkar H, Jin J, Geonzon LC, Bacabac RG, Klein-Nulend J (2021) Cellulose and its derivatives: towards biomedical applications. Cellulose 28(4):1893–1931 10.1007/s10570-020-03674-w

[CR61] Shi X-W, Zheng F, Pan R-f, Wang J, Ding S (2014) Engineering and comparative characteristics of double carbohydrate binding modules as a strength additive for papermaking applications. BioResources 9(2):3117–3131 10.15376/biores.9.2.3117-3131

[CR62] Shiiba H, Hayashi S, Yui T (2013) Molecular dynamics study of carbohydrate binding module mutants of fungal cellobiohydrolases. Carbohyd Res 374:96–102 10.1016/j.carres.2013.04.001 23660003

[CR63] Shoseyov O, Shani Z, Levy I (2006) Carbohydrate binding modules: biochemical properties and novel applications. Microbiol Mol Biol R 70(2):283–295 10.1128/MMBR.00028-05 PMC1489539 16760304

[CR64] Sidar A, Albuquerque ED, Voshol GP, Ram AFJ, Vijgenboom E, Punt PJ (2020) Carbohydrate binding modules: diversity of domain architecture in amylases and cellulases from filamentous microorganisms. Front Bioeng Biotechnol 8:871 32850729 10.3389/fbioe.2020.00871 PMC7410926

[CR65] Singh S, Hinkley T, Nugen SR, Talbert JN (2019) Colorimetric detection of *Escherichia coli* using engineered bacteriophage and an affinity reporter system. Anal Bioanal Chem 411(27):7273–7279 31511947 10.1007/s00216-019-02095-4 PMC7241434

[CR66] Togo Y, Nakashima K, Mwandira W, Kawasaki S (2020) A novel metal adsorbent composed of a hexa-histidine tag and a carbohydrate-binding module on cellulose. Anal Sci 36(4):459–464 31866603 10.2116/analsci.19P356

[CR67] Tormo J, Lamed R, Chirino AJ, Morag E, Bayer EA, Shoham Y, Steitz TA (1996) Crystal structure of a bacterial family-III cellulose‐binding domain: a general mechanism for attachment to cellulose. EMBO J 15(21):5739–5751 8918451 10.1002/j.1460-2075.1996.tb00960.x PMC452321

[CR68] Van Tilbeurgh H, Tomme P, Claeyssens M, Bhikhabhai R, Pettersson G (1986) Limited proteolysis of the cellobiohydrolase I from *Trichoderma reesei*: separation of functional domains. FEBS Lett 204(2):223–227 10.1016/0014-5793(86)80816-X

[CR69] Wang S, Cui G-Z, Song X-F, Feng Y, Cui Q (2012) Efficiency and stability enhancement of cis-epoxysuccinic acid hydrolase by fusion with a carbohydrate binding module and immobilization onto cellulose. Appl Biochem Biotechnol 168(3):708–717 22843080 10.1007/s12010-012-9811-8

[CR71] Wang Y, Wang X, Xie Y, Zhang K (2018) Functional nanomaterials through esterification of cellulose: a review of chemistry and application. Cellulose 25(7):3703–3731 10.1007/s10570-018-1830-3

[CR72] Wang Z, Qi J, Hinkley TC, Nugen SR, Goddard JM (2021) Recombinant lactase with a cellulose binding domain permits facile immobilization onto cellulose with retained activity. Food Bioprod Process 126:207–214 10.1016/j.fbp.2021.01.010

[CR70] Wang X, Jiang Y, Liu H, Yuan H, Huang D, Wang T (2023) Research progress of multi-enzyme complexes based on the design of scaffold protein. Bioresour Bioprocess 10(1):72 38647916 10.1186/s40643-023-00695-8 PMC10992622

[CR73] Wolfberger A, Petritz A, Fian A, Herka J, Schmidt V, Stadlober B, Kargl R, Spirk S, Griesser T (2015) Photolithographic patterning of cellulose: a versatile dual-tone photoresist for advanced applications. Cellulose 22(1):717–727 26412951 10.1007/s10570-014-0471-4 PMC4579862

[CR74] Xiao Q, Han J, Jiang C, Luo M, Zhang Q, He Z, Hu J, Wang G (2020) Novel fusion protein consisting of metallothionein, cellulose binding module, and superfolder GFP for lead removal from the water decoction of traditional Chinese medicine. ACS Omega 5(6):2893–2898 32095711 10.1021/acsomega.9b03739 PMC7034022

[CR75] Xu GY, Ong E, Gilkes NR, Kilburn DG, Muhandiram DR, Harris-Brandts M, Carver JP, Kay LE, Harvey TS (1995) Solution structure of a cellulose-binding domain from *Cellulomonas fimi* by nuclear magnetic resonance spectroscopy. Biochemistry 34(21):6993–7009 7766609 10.1021/bi00021a011

[CR76] Yang F, Jin ES, Zhu Y, Wu S, Zhu W, Jin Y, Song J (2015) A mini-review on the applications of cellulose-binding domains in lignocellulosic material utilizations. BioRes 10(3):6081–6094 10.15376/biores.10.3.Yang

[CR77] Yang JM, Kim KR, Jeon S, Cha HJ, Kim CS (2021) A sensitive paper-based lateral flow immunoassay platform using engineered cellulose-binding protein linker fused with antibody-binding domains. Sens Actuat B-Chem 329:129099 10.1016/j.snb.2020.129099

[CR78] You C, Zhang Y-HP (2013) Self-assembly of synthetic metabolons through synthetic protein scaffolds: one-step purification, co-immobilization, and substrate channeling. ACS Synth Biol 2(2):102–110 23656373 10.1021/sb300068g

[CR79] Żebrowska J, Mucha P, Prusinowski M, Krefft D, Żylicz-Stachula A, Deptuła M, Skoniecka A, Tymińska A, Zawrzykraj M, Zieliński J, Pikuła M, Skowron PM (2024) Development of hybrid biomicroparticles: cellulose exposing functionalized fusion proteins. Microb Cell Fact 23(1):81 38481305 10.1186/s12934-024-02344-x PMC10938831

[CR80] Zhang Y, Chen S, Wu J, Chen J (2012) Enzymatic surface modification of cellulose acetate fibre by cutinase-CBM (carbohydrate-binding module) fusion proteins. Biocatal Biotransfor 30(2):184–189 10.3109/10242422.2011.638713

